# Multiple cytokine analysis based on QuantiFERON-TB gold plus in different tuberculosis infection status: an exploratory study

**DOI:** 10.1186/s12879-023-08943-0

**Published:** 2024-01-02

**Authors:** Lifan Zhang, Zhengrong Yang, Fengying Wu, Qiping Ge, Yueqiu Zhang, Dongyu Li, Mengqiu Gao, Xiaoqing Liu

**Affiliations:** 1 Division of Infectious Diseases, Department of Internal medicine, State Key Laboratory of Complex Severe and Rare Disease, Peking Union Medical College Hospital, Chinese Academy of Medical Sciences and Peking Union Medical College, Beijing, China; 2 Clinical Epidemiology Unit, Peking Union Medical College, International Clinical Epidemiology Network, Beijing, China; 3 Center for Tuberculosis Research, Chinese Academy of Medical Sciences and Peking Union Medical College, Beijing, China; 4 Beijing Chest Hospital, Capital Medical University/Beijing Tuberculosis and Thoracic Tumor Research Institute, Beijing, China; 5 4+4 Medical Doctor Program, Chinese Academy of Medical Sciences and Peking Union Medical College, Beijing, China

**Keywords:** Tuberculosis, Cytokines, QFT-Plus, IP-10, IL-1Ra

## Abstract

**Background:**

More efficient and convenient diagnostic method is a desperate need to reduce the burden of tuberculosis (TB). This study explores the multiple cytokines secretion based on QuantiFERON-TB Gold Plus (QFT-Plus), and screens for optimal cytokines with diagnostic potential to differentiate TB infection status.

**Methods:**

Twenty active tuberculosis (ATB) patients, fifteen patients with latent TB infection (LTBI), ten patients with previous TB and ten healthy controls (HC) were enrolled. Whole blood samples were collected and stimulated by QFT-Plus TB1 and TB2 antigens. The levels of IFN-γ, TNF-α, IL-2, IL-6, IL-5, IL-10, IP-10, IL-1Ra, CXCL-1 and MCP-1 in supernatant were measured by Luminex bead-based multiplex assays. The receiver operating characteristic curve was used to evaluate the diagnostic accuracy of cytokine for distinguishing different TB infection status.

**Results:**

After stimulation with QFT-Plus TB1 and TB2 antigens, the levels of all cytokines, except IL-5 in TB2 tube, in ATB group were significantly higher than that in HC group. The levels of IL-1Ra concurrently showed the equally highest AUC for distinguishing TB infection from HC, followed by the levels of IP-10 in both TB1 tube and TB2 tube. Moreover, IP-10 levels displayed the largest AUC for distinguishing ATB patients from non-ATB patients. Meanwhile, the levels of IP-10 also demonstrated the largest AUC in both TB1 tube and TB2 tube for distinguishing ATB patients from LTBI.

**Conclusions:**

In addition to conventional detection of IFN-γ, measuring IP-10 and IL-1Ra based on QFT-Plus may have the more tremendous potential to discriminate different TB infection status.

**Supplementary Information:**

The online version contains supplementary material available at 10.1186/s12879-023-08943-0.

## Introduction

Before the COVID-19 pandemic, tuberculosis (TB) was the infectious disease with the most deaths caused by a single pathogen. China has the third largest burden of TB in the world, with an estimated annually new active tuberculosis (ATB) patients exceeding 780,000 [1], latent tuberculosis infection (LTBI) rate is as high as 20%, with nearly 300 million LTBI [2]. The incidence of catastrophic expenditure caused by pulmonary TB diagnosis and treatment is as high as 20 -70% [3]. Nearly 50% of the cost occurred before treatment [4]. Early rapid and accurate diagnosis of ATB can reduce the economic burden of patients, reduce the mortality and transmission risk, which has significant economic and social benefits.

TB diagnosis faces many challenges. In China, only 50% of pulmonary TB and 13% of extrapulmonary TB can be diagnosed by etiology [1, 5]. Interferon-γ (IFN-γ) release assays (IGRAs), which is currently used in clinical practice, detects IFN-γ released after stimulation by Mtb specific antigen to diagnose TB infection. It has high sensitivity, which is helpful to exclude ATB, but has limited effect in the diagnosis of ATB [6] and also cannot distinguish ATB and LTBI [7]. However, In the process of clinical diagnosis and treatment, especially in general hospitals, many patients’ conditions are complex and need to be identified with ATB. Under the background of high LTBI rate (~ 20%) in China, how to quickly and accurately diagnose ATB, distinguish ATB from LTBI, and give corresponding anti-TB treatment or TB preventive treatment in a large number of suspected patients who cannot obtain etiological evidence is an urgent clinical problem to be solved.

QuantiFERON-TB Gold Plus (QFT-Plus) as the latest generation of IGRAs, the TB1 tube includes early secreting antigen target-6 (ESAT-6) and culture filtrate protein-10 (CFP-10)-derived peptides, designed to show cell-mediated immune responses from CD4 T cells. Meanwhile, the TB2 tube contains new peptides capable of eliciting IFN-γ production by both CD4 and CD8 T-cell responses. The novelty of QFT-Plus lies in the fact that it could elicit an additional response from CD8 T cells, thus collecting a broader response from T-cell subsets compared to QFT-GIT [8–10]. Prior studies have shown a significant variation of antigen-specific CD8 T cells producing IFN-γ and other cytokines exist in different TB infection status [11–13]. However, although IFN-γ is necessary for host immunity against TB, this cytokine alone is insufficient to provide protection, thus only measuring IFN-γ response by IGRAs may leave out other key molecules in TB infection diagnosis. Other cytokines associated with T cell functional diversity and engaged into underlying pathogenesis of TB infection should be necessarily taken into consideration as candidate biomarkers for better clinical TB diagnosis and assessment of disease status [14].

Mounting data suggested that the numerous cytokines including interferons, interleukins, tumor necrosis factors and chemokines serve as important mediators in cellular immune responses to TB infection [15–18]. Several studies reported that interleukin (IL)-1 receptor antagonist (IL-1Ra), IL-2, IFN-γ-inducible protein of 10 kDa (IP-10), monocyte chemotactic protein (MCP)-2, tumor necrosis factor superfamily member 14, IL-5, IL-10 and MCP-1 in different measurement methods had promising diagnostic performance for TB infection, including both ATB and LTBI [19–25]. Moreover, people with previous TB history have elevated risk of TB reactivation as the same as LTBI [26, 27]. The immune response of multiple cytokines stimulated by QFT-Plus antigens among ATB, LTBI, people with previous TB history and healthy people remained unclear. Therefore, this study was performed to explore the differences of multiple cytokines secretion based on QFT-Plus antigens stimulation, and to screen for optimal cytokines with potential to differentiate different TB infection status.

## Methods

### Study design and subjects

In this research, we conducted a cross-sectional study. Details regarding the inclusion and exclusion criteria were displayed in Table 1, ATB patients were recruited from the Peking Union Medical College Hospital and Beijing Chest Hospital between February and April 2022. Meanwhile, we enrolled LTBI with IGRA-positive (named HCW + IGRAs(LTBI) ) and previous TB patients from healthcare workers (HCWs) in Beijing Chest Hospital, and freshman of Peking Union Medical College were selected as healthy control (HC) as no abnormality was noticed during admission medical examination.

**Table 1:** The inclusion and exclusion criteria for enrolled groups

Diagnostic category	Inclusion criteria	Exclusion criteria
ATB	a) Positive results of *Mtb* culture/GeneXpert/PCR/ acid-fast staining of sputum or other samples AND b) Clinical manifestations (fever, cough, chest pain, night sweats, and weight loss, etc.) AND c) Imaging characteristics suggesting ATB AND d) Untreated with anti-tuberculosis treatment.	Patients with hematologic malignancies, HIV infection, during pregnancy or breastfeeding.
LTBI	a) Positive results of IGRAs AND b) Laboratory results and imaging characteristics not suggesting ATB AND c) Imaging in six months not suggesting evidence of previous TB.	
Previous TB	a) Laboratory results and imaging characteristics suggesting evidence of previous TB AND b) Previous TB history.	
HC	a) Negative results of IGRAs AND b) Clinical manifestations and imaging in six months not suggesting ATB and evidence of previous TB AND c) No previous history of ATB AND d) Denial of history of TB exposure.	

ATB, active tuberculosis; *Mtb*, Mycobacterium tuberculosis; LTBI, latent tuberculosis infection; IGRAs, interferon-γ release assays; HC, healthy control

First, the main difference between the LTBI group and the previous TB group is whether there is evidence of previous tuberculosis, including old tuberculosis lesions reported by chest imaging and a clear history of tuberculosis diagnosed by the hospital. Second, the IGRA results of LTBI group are all positive, while the IGRA results of previous TB group may be positive or negative. In this study, 10 cases of HCW with previous TB were included, of which 8 cases were tested for IGRA, 4 cases were positive and 4 cases were negative. Re-analysis of the data showed that there was no change in the results (Supplementary Figs. 2–5, Supplementary Table 2). Relevant figures/tables have been seen as supplementary materials.

**Fig. 1:**
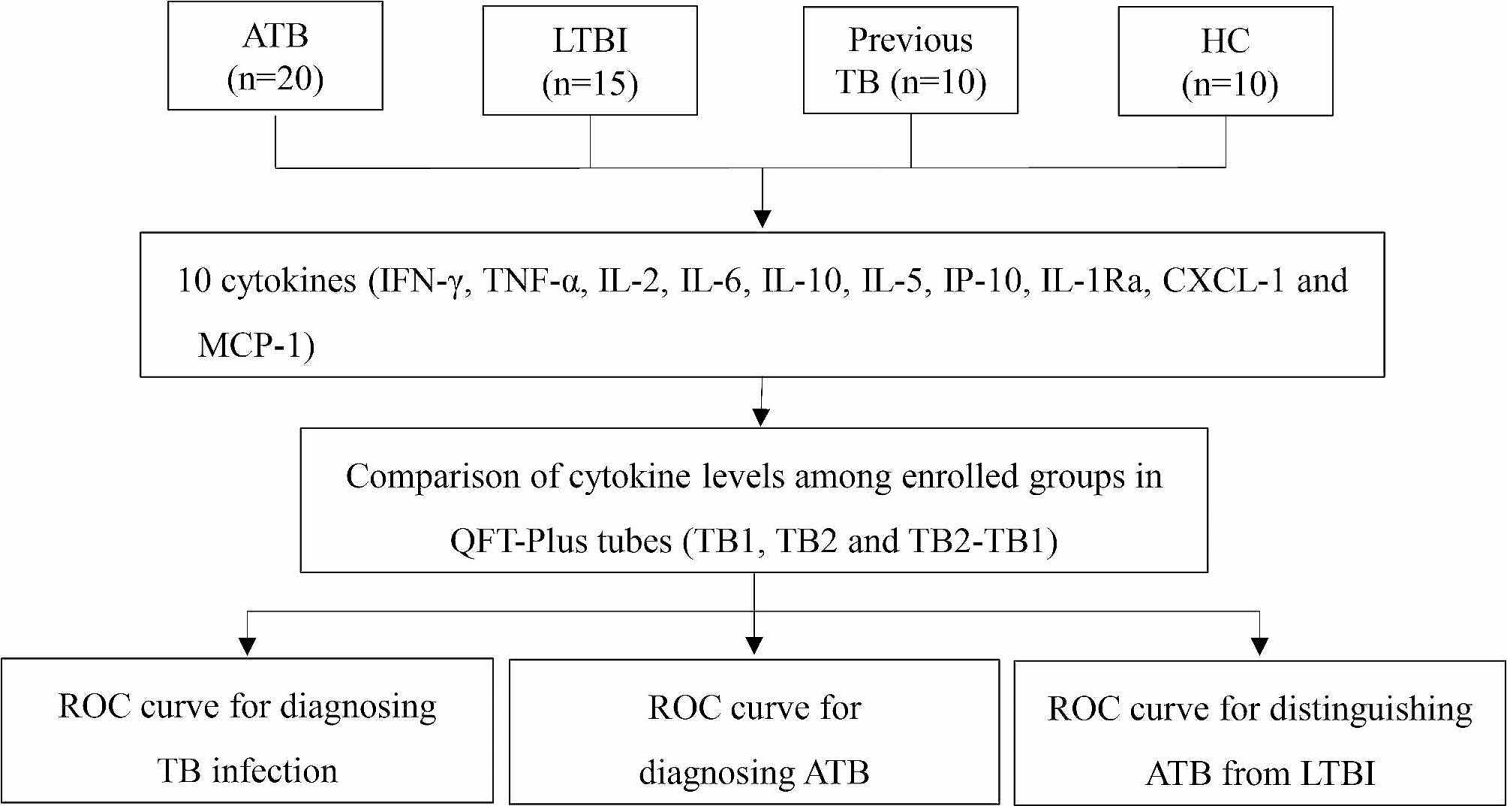
Study flow chart

The demographic characteristics and laboratory examinations of the study subjects were listed in Table 2. Later, the level of cytokines (IFN-γ, TNF-α, IL-2, IL-6, IL-10, IL-5, IP-10, IL-1Ra, CXCL-1 and MCP-1) were tested by Luminex assays in QFT-Plus tubes for each participant and were compared among enrolled groups to evaluate their capabilities in distinguishing different TB infection status. The study flow chart as shown in Fig. 1. Our study was approved by the Ethics Committee of PUMCH (No: ZS-3415) and written informed consent forms were obtained in accordance with the Declaration of Helsinki before collecting blood samples.

**Table 2:** Baseline characteristics of the study subjects

Characteristics	ATB (N = 20)	LTBI (N = 15)	Previous TB (N = 10)	HC (N = 10)
Age, years	50.3 ± 16.13	44.9 ± 7.22	47.4 ± 6.57	26.4 ± 2.41
Male	14 (70)	2 (13.3)	3 (30)	4 (40)
BMI, kg/m^2^	22.7 ± 3.47	24.5 ± 2.64	24.9 ± 7.56	20.8 ± 2.45
Site				
Lung TB	18 (90)	/	/	/
Liver TB	1	/	/	/
Bone TB	1	/	/	/
Evidence of previous TB	10 (50)	0	10 (100)	0
Presence of TB history	0	0	10 (100)	0
Diabetes mellitus	6 (30)	0	0	0
Blood test				
White blood cell count, 10^9^ /L	7.38 (5.47, 8.60)	6.24 (5.84, 7.17)	5.75 (5.30, 6.36)	6.04 (5.63, 6.64)
Neutrophil count, 10^9^ /L	4.90 (3.63, 6.29)	3.76 (3.02, 5.30)	3.40 (3.09, 4.00)	4.02 (3.53, 4.22)
Lymphocyte count, 10^9^ /L	1.54 (1.12, 1.67)	1.95 (1.58, 2.45)	1.81 (1.70, 1.87)	1.67 (1.53, 1.92)
Platelet count, 10^9^ /L	299 (235, 378)	271 (217, 313)	276 (189, 301)	229 (217, 256)
Hemoglobin, g/L	118 (111, 134)	136 (116, 143)	147 (139, 151)	124 (118, 131)

Values are presented as N (%) or mean ± SD or median, IQR. N, number; SD, Standard deviation; IQR, interquartile range; / means no data

### QFT-Plus

QFT-Plus tests were performed in accordance with the manufacturer’s instructions. Briefly, heparin anticoagulant whole blood which need to handle as soon as possible within 6 h was added into each QFT-Plus blood collection tube (QIAGEN, Dusseldorf, Germany) and incubated at 37 C for 24 h with TB-specific antigens, including TB1 antigen and TB2 antigen, a mitogen as a positive control, and without stimulation as a negative control (Nil). Further, the QFT-Plus tubes were centrifuged 3000 g for 15 min and supernatants have been collected and retained [28].

### Luminex assays for cytokines in the supernatants of QFT-Plus

The levels of cytokines in the QFT-Plus supernatants were measured by a custom Human Premixed Multi-Analyte Kit (Bio-Rad Laboratories, Hercules, CA, USA) which could simultaneously detect 10 cytokines (IFN-γ, TNF-α, IL-2, IL-6, IL-10, IL-5, IP-10, IL-1Ra, CXCL-1 and MCP-1). Cytokine-specific antibodies were pre-coated onto magnetic microparticles embedded with fluorophores at set ratios for each unique microparticle region. Microparticles, standards and samples were pipetted into wells and the immobilized antibodies would therefore bind to the cytokines. After washing away any unbound substances, a biotinylated antibody cocktail specific to the cytokines was added to each well. After removing any unbound biotinylated antibody, streptavidin-phycoerythrin conjugate (Streptavidin-PE), which binds to the biotinylated antibody, was added to each well. Finally, after washing off unbound Streptavidin-PE, the microparticles were resuspended in buffer. Final parameters were detected using the Luminex 200 Multiplexing Instrument with xPONENT software (Luminex, Austin, TX, USA) and analyzed using Milliplex Analyst software (Millipore Sigma, Billerica, MA, USA) based on the manufacturer’s instructions [29].

### Statistical analysis

Statistical analysis was performed using SPSS 26.0 (IBM, Armonk, NY, USA) and GraphPad Prism 9 (GraphPad software, San Diego, CA). The Kolmogorov–Smirnov test was adopted to examine whether the continuous variables followed a normal distribution. The variables with normal distribution were denoted as the mean ± standard deviation, whereas the variables with non-normal distribution were denoted as the median and interquartile range. Ordinary one-way ANOVA test with Tukey test and Kruskal-Wallis test with Dunn’s test were used to compare the distribution of variables across four groups and pairwise comparisons between each independent group. P values were calculated to determined which groups were statistically significantly different. Receiver operating characteristic (ROC) curves were used to distinguish different TB infection status by comparing each cytokine level and the area under the curve (AUC) were calculated. A two-tailed P-value of less than 0.05 was considered statistically significant.

## Results

### Comparison of cytokine levels among enrolled groups in QFT-Plus TB1 and TB2 tubes

**Fig. 2:**
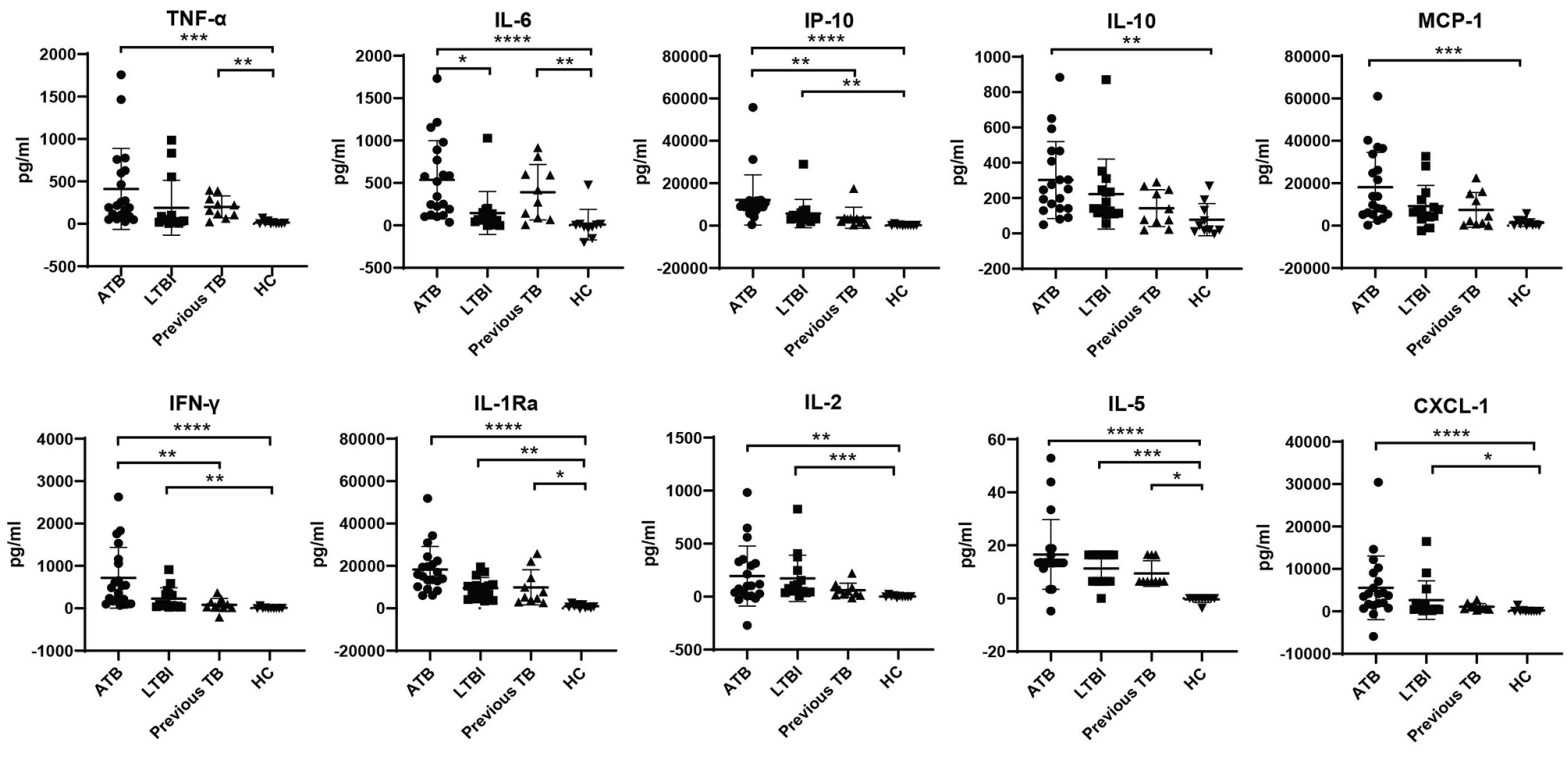
The levels of 10 cytokines in QFT-Plus TB1 antigen tubes with the levels in the corresponding Nil tubes subtracted in the ATB, LTBI, previous TB and HC groups. *P < 0.05, **P < 0.01, ***P < 0.001, ****P < 0.0001. Bars represent mean values, and error bars represent SD

Different cytokine levels in both TB1 and TB2 tubes were subtracted with those in the Nil tubes. In the TB1 tube, the levels of all cytokines showed significant differences among four groups. Notably, the levels of TNF-α, IL-6, IP-10, IL-10, MCP-1, IFN-γ, IL-1Ra, IL-2, IL-5 and CXCL-1 in ATB patients were significantly increased when comparing with these in HC. Moreover, the levels of IP-10, IFN-γ, IL-1Ra, IL-2, IL-5 and CXCL-1 in HCW + IGRAS(LTBI) were also significantly increased when comparing with these in HC. Nevertheless, TNF-α, IL-6 IL-1Ra and IL-5 were the only four cytokines that was elevated in patients with previous TB infection comparing with HC. Furthermore, we found that the level of IL-6 in ATB patients was increased in comparison with HCW + IGRAS(LTBI) the level of IP-10 and IFN-γ in ATB patients was increased in comparison with previous TB (Fig. 2).

**Fig. 3:**
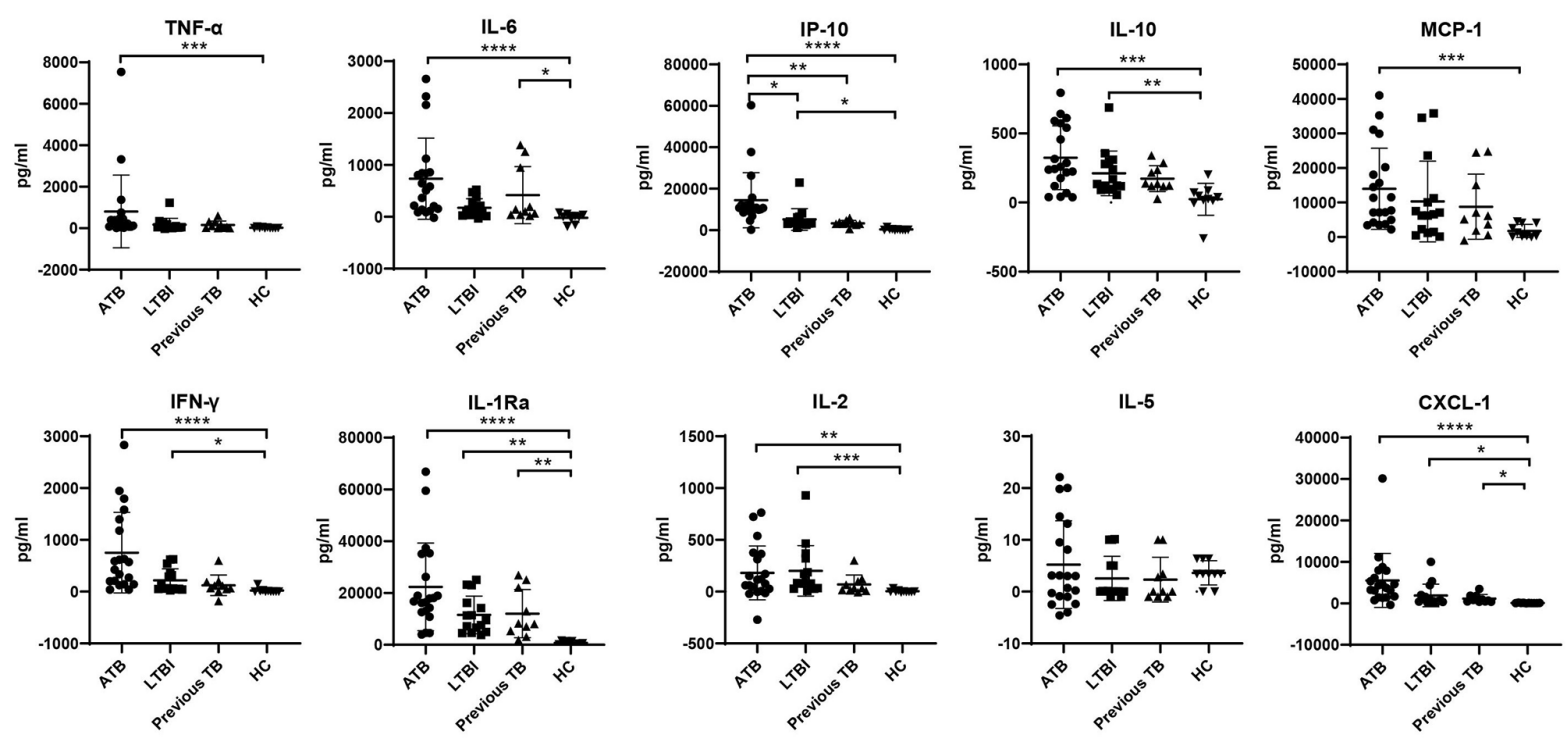
The levels of 10 cytokines in QFT-Plus TB2 antigen tubes with the levels in the corresponding Nil tubes subtracted in the ATB, LTBI, previous TB and HC groups. *P < 0.05, **P < 0.01, ***P < 0.001, ****P < 0.0001. Bars represent mean values, and error bars represent SD

Different patterns were observed in the TB2 tubes, where the levels of all cytokines, except IL-5, showed significant differences among enrolled four groups. The levels of TNF-α, IL-6, IP-10, IL-10, MCP-1, IFN-γ, IL-1Ra, IL-2 and CXCL-1 in ATB patients were significantly elevated when comparing with these in HC. Moreover, the levels of IP-10, IL-10, IFN-γ, IL-1Ra, IL-2 and CXCL-1 in HCW + IGRAS(LTBI) were also significantly increased when comparing with these in HC. However, IL-6, IL-1Ra, and CXCL-1 were the only three cytokines that was elevated in patients with previous TB infection comparing with HC. Interestingly, we found that the level of IP-10 in ATB patients was increased in comparison with both HCW + IGRAS(LTBI) and previous TB patients, however, no significant difference in IP-10 was observed between HCW + IGRAS(LTBI) and previous TB patients (Fig. 3).

**Fig. 4:**
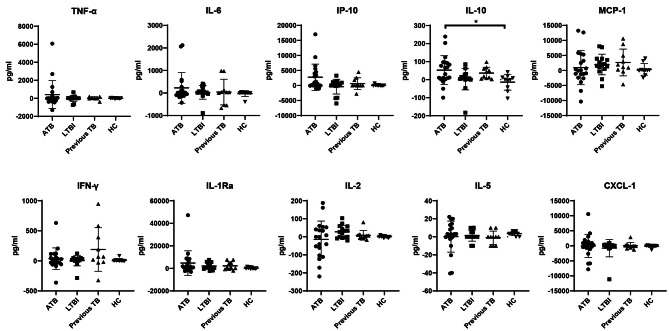
The levels of 10 cytokines in QFT-Plus TB2 antigen tubes with the levels in the corresponding TB1 antigen tubes subtracted in the ATB, LTBI, previous TB and HC groups. *P < 0.05. Bars represent mean values, and error bars represent SD

We further explored secretion patterns of TB2 antigen tubes after subtracting corresponding the cytokine levels in TB1 tubes in four different groups. Interestingly, with the exception of IL-10 level, there was an apparent rise in ATB patients when comparing with HC, and we haven’t yet found any significant discrepancy of the other cytokines among four groups (Fig. 4).

### ROC curves for cytokine levels for distinguishing TB infection from HC

**Fig. 5:**
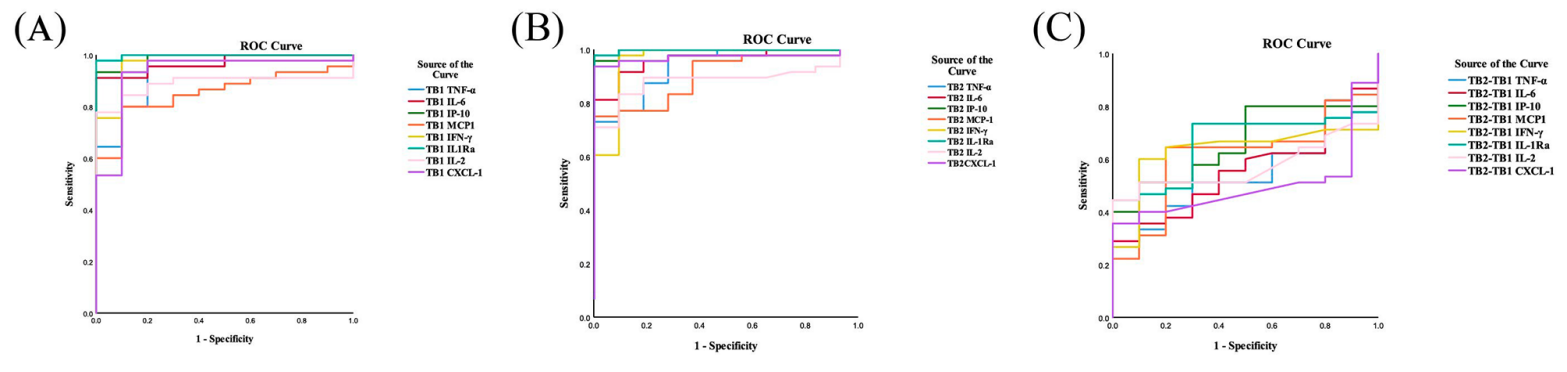
ROC curve of cytokines in QFT-Plus TB1 and TB2 antigen tubes for diagnosing TB infection. (**A**) ROC curve of the accuracy of cytokine levels stimulated by antigen in TB1 tube of QFT-Plus with the levels in the corresponding Nil tubes subtracted for differentiating TB infection from HC. (**B**) ROC curve of the accuracy of cytokine levels stimulated by antigen in TB2 tube of QFT-Plus with the levels in the corresponding Nil tubes subtracted for differentiating TB infection from HC. (**C**) ROC curve of the accuracy of cytokine levels stimulated by antigen in TB2 tube of QFT-Plus with the levels in the corresponding TB1 tubes subtracted for differentiating TB infection from HC. Lines of different colors corresponding to matched cytokines

All participants enrolled in this study were subsequently divided into HC group and TB infection group, including ATB, HCW + IGRAS(LTBI) and previous TB patients. In order to distinguish TB infection from HC, we generated ROC curves as they could determine the best cutoff value of each cytokine levels. Among the ROC curves using cytokine levels in TB1 (Fig. 5A) and TB2 (Fig. 5B) tubes separately subtracted with the corresponding cytokine levels in the Nil tube and in TB2 tube minus TB1 tube (Fig. 5C), the levels of IL-1Ra concurrently showed the equally highest AUC in both the TB1 and TB2 tubes (AUC = 0.998), and demonstrated they had the best diagnostic accuracy for distinguishing TB infection from HC, followed by the levels of IP-10 in both TB1 tube (AUC = 0.987) and TB2 tube (AUC = 0.996). Intriguingly, the levels of TNF-α, IL-6, IFN-γ and CXCL-1 in TB1 and TB2 tubes had almost equal diagnostic accuracy to IL-1Ra and IP-10 as their AUC were all greater than 0.9. However, the levels of MCP-1 and IL-2 in TB1 and TB2 tubes had a little lower AUC than other cytokines analyzed. Moreover, there was no significance of above cytokines in TB2-TB1 for distinguishing TB infection from HC (Table 3). Considering the level of IL-10 in TB2-TB1 was an apparent rise in ATB patients comparing to HC, ROC curves were plotted and showed a good accuracy of IL-10 level in TB2-TB1 for differentiating TB infection from HC (AUC = 0.742) (Supplementary Fig. 1A, Supplementary Table 1).

**Table 3:** AUCs of cytokines in each antigen tube for diagnosing TB infection

Parameter	TNF-α	IL-6	IP-10	MCP-1	IFN-γ	IL-1Ra	IL-2	CXCL-1
TB1-Nil								
AUC (95%CI)	0.931 (0.852-1.000)	0.969 (0.929-1.000)	0.987 (0.963-1.000)	0.853 (0.751–0.956)	0.956 (0.894-1.000)	0.998 (0.991-1.000)	0.889 (0.801–0.977)	0.929 (0.834-1.000)
*p*	< 0.001	< 0.001	< 0.001	0.001	< 0.001	< 0.001	< 0.001	< 0.001
TB2-Nil								
AUC (95%CI)	0.929 (0.852-1.000)	0.958 (0.906-1.000)	0.996 (0.984-1.000)	0.889 (0.798–0.980)	0.956 (0.875-1.000)	0.998 (0.991-1.000)	0.870 (0.775–0.965)	0.969 (0.923-1.000)
*p*	< 0.001	< 0.001	< 0.001	0.002	< 0.001	< 0.001	< 0.001	< 0.001
TB2-TB1								
AUC (95%CI)	0.533 (0. 379-0. 688)	0. 559 (0.399–0.719)	0.662 (0.517–0.808)	0.611 (0.449–0.773)	0.633 (0.485–0.781)	0.660 (0.517–0.803)	0.572 (0.430–0.715)	0.497 (0.342–0.651)
*p*	0.743	0.563	0.111	0.275	0.190	0.116	0.478	0.974

AUC, area under the curve; CI, confidence interval

### ROC curves for cytokine levels for distinguishing ATB from non-ATB

**Fig. 6:**
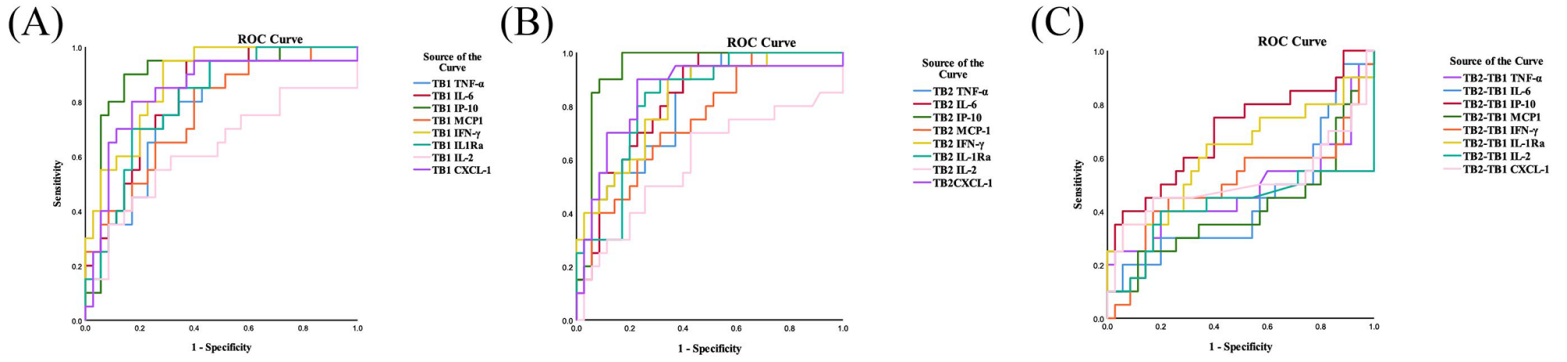
ROC curve of cytokines in QFT-Plus TB1 and TB2 antigen tubes for diagnosing ATB. (**A**) ROC curve of the accuracy of cytokine levels stimulated by antigen in TB1 tube of QFT-Plus with the levels in the corresponding Nil tubes subtracted for differentiating ATB from inactive TB. (**B**) ROC curve of the accuracy of cytokine levels stimulated by antigen in TB2 tube of QFT-Plus with the levels in the corresponding Nil tubes subtracted for differentiating ATB from inactive TB. (**C**) ROC curve of the accuracy of cytokine levels stimulated by antigen in TB2 tube of QFT-Plus with the levels in the corresponding TB1 tubes subtracted for differentiating ATB from inactive TB. Lines of different colors corresponding to matched cytokines

These HCW + IGRAS(LTBI), previous TB patients and HC were further amalgamated into non-ATB patients, The ROC curves were generated by the levels of cytokines stimulated with antigens in TB1 (Fig. 6A) and TB2 (Fig. 6B) tubes separately subtracted with the corresponding cytokine levels in the Nil tube and in TB2 tube minus TB1 tube (Fig. 6C). We found that the level of IP-10 showed the largest AUC in both TB1 tube (AUC = 0.897) and TB2 tube (AUC = 0.94). Interestingly, IP-10 level in TB2-TB1 also indicated a good accuracy for distinguishing ATB from non-ATB (AUC = 0.699) Furthermore, the levels of TNF-α, IL-6, IFN-γ, IL-1Ra and CXCL-1 in TB1 and TB2 tubes also had excellent diagnostic accuracy for distinguishing ATB patients from non-ATB patients as their AUC around 0.8, but AUC of MCP-1 was lower than above cytokines. Nevertheless, the levels of IL-2 in TB1 and TB2 tubes had limited diagnostic accuracy, as well as cytokines in TB2-TB1 except IP-10 (Table 4). As the level of IL-10 in TB2-TB1 was an apparent rise in ATB patients, there was a satisfied accuracy of IL-10 level in TB2-TB1 for differentiating ATB patients from non-ATB (AUC = 0.669) (Supplementary Fig. 1B, Supplementary Table 1).

**Table 4:** AUCs of cytokines in each antigen tube for diagnosing ATB

Parameter	TNF-α	IL-6	IP-10	MCP-1	IFN-γ	IL-1Ra	IL-2	CXCL-1
TB1-Nil								
AUC (95%CI)	0.78 (0.66–0.90)	0.819 (0.709–0.928)	0.897 (0.802–0.992)	0.760 (0.631–0.889)	0.879 (0.792–0.965)	0.807 (0.693–0.921)	0.623 (0.456–0.790)	0.840 (0.72–0.96)
*p*	0.001	< 0.001	< 0.001	0.001	< 0.001	< 0.001	0.132	< 0.001
TB2-Nil								
AUC (95%CI)	0.801 (0. 687-0.916)	0.829 (0.723–0.934)	0.94 (0.873-1.000)	0.75 (0.62–0.88)	0.823 (0.712–0.933)	0.821 (0.711–0.932)	0.592 (0.423–0.761)	0.848 (0.731–0.965)
*p*	< 0.001	< 0.001	< 0.001	0.002	< 0.001	< 0.001	0.259	< 0.001
TB2-TB1								
AUC (95%CI)	0. 481 (0.300-0.663)	0.443 (0.274–0.612)	0.699 (0.546–0.851)	0.409 (0.236–0.581)	0.500 (0.322–0.678)	0.621 (0.453–0.790)	0.419 (0.230–0.607)	0.509 (0.321–0.698)
*p*	0.820	0.484	0.015	0.263	1.000	0.137	0.319	0.909

AUC, area under the curve; CI, confidence interval

### ROC curves for cytokine levels for distinguishing ATB from HCW + IGRAS(LTBI)

**Fig. 7:**
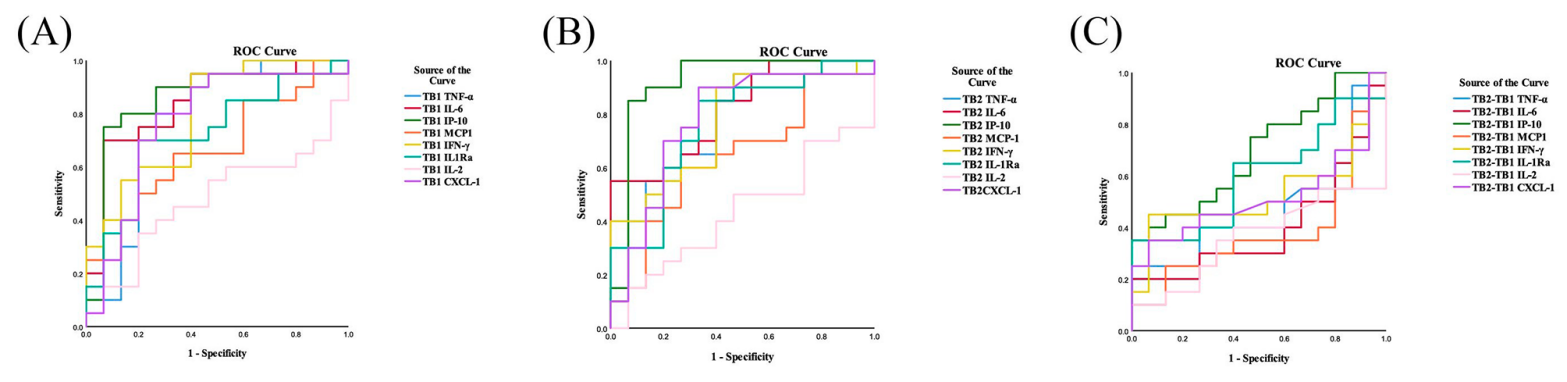
ROC curve of cytokines in QFT-Plus TB1 and TB2 antigen tubes for distinguishing ATB from LTBI. (**A**) ROC curve of the accuracy of cytokine levels stimulated by antigen in TB1 tube of QFT-Plus with the levels in the corresponding Nil tubes subtracted for differentiating ATB from LTBI. (**B**) ROC curve of the accuracy of cytokine levels stimulated by antigen in TB2 tube of QFT-Plus with the levels in the corresponding Nil tubes subtracted for differentiating ATB from LTBI. (**C**) ROC curve of the accuracy of cytokine levels stimulated by antigen in TB2 tube of QFT-Plus with the levels in the corresponding TB1 tubes subtracted for differentiating ATB from LTBI. Lines of different colors corresponding to matched cytokines

Finally, diagnostic accuracy of cytokine levels in TB1 (Fig. 7A) and TB2 (Fig. 7B) tubes subtracted with the cytokine levels in the Nil tube and in TB2 tube minus TB1 tube (Fig. 7C) for distinguishing ATB from HCW + IGRAS(LTBI) was evaluated by ROC curves. As was expected, the level of IP-10 showed the largest AUC in both TB1 tube (AUC = 0.850) and TB2 tube (AUC = 0.920), followed by the level of IL-6 in TB1 tube (AUC = 0.843) and the level of IL-6 in TB2 tube (AUC = 0.817). In addition, the levels of TNF-α, IL-1Ra and CXCR1 in TB1 and IL-Ra in TB2 tubes had decreased diagnostic accuracy in comparison with IFN-γ for distinguishing ATB patients from HCW + IGRAS(LTBI). However, besides the levels of MCP-1 and IL-2 in TB1 and TB2 tubes, the levels of the above cytokines in TB2-TB1 included IL-10 had also no diagnostic efficiency for differentiating ATB patients from HCW + IGRAS(LTBI) (Table 5, Supplementary Fig. 1C, Supplementary Table 1).

**Table 5:** AUCs of cytokines in each antigen tube for distinguishing ATB from LTBI

Parameter	TNF-α	IL-6	IP-10	MCP-1	IFN-γ	IL-1Ra	IL-2	CXCL-1
TB1-Nil								
AUC (95%CI)	0.780 (0.605–0.955)	0.843 (0.707–0.980)	0.850 (0.704–0.996)	0.667 (0.487–0.847)	0.793 (0.641–0.946)	0.723 (0.551–0.896)	0.477 (0.282–0.671)	0.767 (0.594–0.940)
*p*	0.005	0.001	< 0.001	0.096	0.003	0.026	0.816	0.008
TB2-Nil								
AUC (95%CI)	0.780 (0.622–0.938)	0.817 (0.679–0.955)	0.920 (0.808-1.000)	0.650 (0.463–0.837)	0.773 (0.616–0.931)	0.763 (0.599–0.927)	0.433 (0.242–0.625)	0.778 (0.611–0.945)
*p*	0.005	0.002	< 0.001	0.134	0.006	0.008	0.505	0.005
TB2-TB1								
AUC (95%CI)	0.475 (0.278–0.672)	0.403 (0.210–0.597)	0.693 (0.520–0.867)	0.380 (0.187–0.573)	0.527 (0.328–0.726)	0.600 (0.410–0.790)	0.363 (0.177–0.549)	0.517 (0.319–0.714)
*p*	0.803	0.334	0.053	0.230	0.790	0.317	0.172	0.868

AUC, area under the curve; CI, confidence interval

## Discussion

In this study, we identified that the levels of all cytokines in TB1 and TB2 tubes showed significant differences among four groups, except the IL-5 in TB2 tube after stimulation with TB1 and TB2 antigens. However, the values of all cytokines, except IL-10, in TB2 tube minus corresponding values in TB1 tube showed no significant differences among four groups. Furthermore, we demonstrated that the level of IL-1Ra in both TB1 and TB2 tubes in ATB patients were the most significantly elevated parameters in comparison with HC. Moreover, among three TB infection groups (ATB, LTBI, previous TB), we observed that the level of IL-6 in TB1 tube and the level of IP-10 in TB2 tube in ATB patients were higher than these in LTBI, however, no significant distinctions were observed in the levels of IFN-γ or IL-1Ra between ATB and LTBI, despite their *P-*values being close to 0.05. It is plausible to speculate that the relatively small sample size may account for this outcome. Moreover, the level of IP-10 in TB1 and TB2 tubes and the level of IFN-γ in TB1 tube in previous TB patients were decreased when comparing with these in ATB patients, and only the level of IP-10 in TB2 tube in LTBI was higher than that in previous TB patients. Finally, we demonstrated that IL-1Ra may be the most promising biomarker to differentiate TB infection from HC and IP-10 could serve as the most ideal index to differentiate ATB from non-ATB and LTBI.

The aim of this study is to identify biomarkers that can distinguish between various TB infection statuses, including ATB, LTBI, previous TB, and uninfected individuals with no exposure to Mtb. To achieve this, it is essential to establish precise definitions for individuals with different TB infection statuses. Ideally, each group of research subjects should be distinct and independent from one another. For instance, within the HC group, we have selected IGRA-negative freshmen as they are most likely uninfected with Mtb. In the case of HCWs who are IGRA-negative, we must exercise caution due to their continuous exposure to Mtb. We cannot entirely rule out LTBI status for this group since IGRA detection relies on the body’s specific immune response to Mtb antigens. The results may be influenced by an individual’s immune status, potentially leading to false negatives.

Our data showed that mycobacterium tuberculosis (Mtb)-specific IFN-γ, TNF-α, IL-2, IL-6, IL-10, IL-5, IP-10, IL-1Ra, CXCL-1 and MCP-1 responses based on QFT-Plus significantly differed between Mtb-infected (including ATB, LTBI and previous TB) and uninfected healthy populations. Many studies have compared the plasma MCP-1 levels in ATB, LTBI, and HC [22, 30–33], but the conclusions are inconsistent. This inconsistency may derive from individual differences in the participants included or from the differences in the use of MTB-specific antigenic stimulation. In addition, these cytokines were used to further ROC analysis except IL-10 and IL-5, which were removed due to low concentration after Mtb antigen stimulation. The cytokines including IFN-γ, TNF-α, IL-2, IL-6, IP-10, IL-1Ra, CXCL-1 and MCP-1 displayed optimal AUC value, indicating their potential to serve as immunodiagnostic biomarkers for TB infection. Multiple studies have highlighted that Mtb-specific IL-1Ra response is the most consistent parameter. Therefore, this cytokine is widely utilized in whole blood as well as peripheral blood mononuclear cells as alternative biomarkers for IFN-γ to detect Mtb-infected individuals, irrespective of age and sex [34–36]. And our finding was in accordance with these studies, suggesting the levels of IL-1Ra in TB1 and TB2 antigen tubes were regarded as the most promising cytokine for distinguishing TB infection from HC because of their high AUC. The ability of IL-1Ra in differentiating TB infection from HC could imply their significance in TB pathogenesis. Up till now, the role of IL-1Ra in immunity against Mtb has not been fully elucidated. As has been reported before, IL-1Ra is essential in the recruitment of macrophages as well as the production of IFN-γ in the pathogenesis of TB infection [37]. Furthermore, IL-1Ra is also an important mediator of type I IFN-driven susceptibility to TB infection in vivo [38]. In addition, polymorphisms of IL-1Ra is reported capable of altering disease progression in human TB [39]. IL-1Ra can therefore serve as an optimal biomarker for diagnosing TB infection. Moreover, for the first time, our study reveals that Mtb-specific CXCL-1 levels in TB1 and TB2 tubes are significantly increased in Mtb-infected populations and are considered as immunodiagnostic biomarkers of TB infection, indicating an important role for CXCL-1 in immunity to Mtb. This conclusion is supported by a previous study showing that CXCL-1 is critical to neutrophil recruitment into the lungs of Mtb-infected mice during the adaptive immune response [40]. However, a more precise mechanism of these cytokines remains further exploration and would be the major research focus of our team in the future.

Consistent with previous studies [41, 42], our study suggested that the levels of IP-10, TNF-α, IL-6, IFN-γ, IL-1Ra and CXCL-1 in TB1 and TB2 tubes had excellent diagnostic capabilities for distinguishing ATB patients from non-ATB patients (including LTBI and previous TB) and LTBI. Most notably, we demonstrated that IP-10 was the most ideal biomarker for diagnosing ATB, with the higher AUC in discriminating ATB from non-ATB and LTBI than other evaluated parameters in our study. Therefore, the use of the IP-10 level might leads to an improved diagnosis of ATB disease when combined with the currently standard diagnostic methods, including microbiology evidence, molecular tests, and clinical and radiological assessments. Our results showed a higher level of IP-10 was correlated to a higher diagnostic accuracy in ATB patients, consistent with previously published studies which have shown that Mtb antigen-stimulated IP-10 responses have a sensitivity similar to that of QFT-GIT for detecting ATB, thereby highlighting the diagnostic potential of IP-10 in distinguishing between ATB and LTBI [33, 43–45]. IP-10 is expressed in the bronchial epithelium and in inflamed tissue of ATB patients [46, 47] Major functions of IP-10 involves recruitment of activated immune cells, which is necessary for TB protective immunity [48]. In addition, IP-10 may also contribute to the formation and maintenance of granulomas in TB [49]. According to results of this study, IP-10 can serve as a potential diagnostic biomarker of ATB, implying that IP-10 may be a pivotal mediator engaged into activity and progression of TB infection. Nevertheless, the levels of IL-2 in TB1 and TB2 tubes had limited diagnostic accuracy both in differentiating ATB from non-ATB and LTBI. In contrast, some previous studies reported that IL-2 is useful in the discrimination of LTBI from ATB [21, 50]. A meta-analysis also reported that IL-2 had the highest overall accuracy in defining the distinction between ATB and LTBI [24]. This discrepancy with previous studies could derive from the fact that level of IL-2 in the supernatant of QFT-Plus may not be stable and fluctuate among the samples or depend on the assay used to measure IL-2. Further study with stable and accurate assay, or greater sample size study design should be conducted before a conclusion is drawn.

In the QFT-Plus assay, the TB1 antigen tube, which contains the ESAT-6 and CFP-10 peptide antigens to primarily detect the CD4 T cell response; and the TB2 antigen tube, which contains additional shorter peptides from ESAT-6 and CFP-10 to detect both the CD4 and CD8 T cell responses [51]. Therefore, the cytokine levels in TB2 tube minus TB1 tube maybe partly reflected Mtb-specific CD8 T cell response. However, we haven’t found any levels of cytokines variation among different TB infection status except IL-10. This is in accordance to the literatures that the cytokines profile of Mtb-specific CD8 T cells is not significantly different between TB and LTBI subjects [11] and our previous study that the level of IFN-γ in TB2 tube minus TB1 tube between ATB and LTBI is no statistical significant difference [52]. There are several evidences that point to the importance of CD8 T cells involved in immune response to Mtb and control of Mtb infection, yet it mostly depends on cytotoxicity and killing of CD8 T cells to achieve containment of intracellular infection by degranulation and direct contact to recognize and eliminate Mtb-infected cells [53–55]. Although CD8 T cells producing cytokines are present in granulomas, systemic responses in the blood aren’t accurately identical to local T cell responses within granulomas [56]. We showed here that Mtb-specific CD8 T cells produce more IL-10 upon antigen recognition in ATB than HC. This is consistent with previous research that the CD8 T cells were responsible for the majority of IL-10 production Mtb infection [57]. And the level of IL-10 in TB2-TB1 displayed a good accuracy to diagnose TB infection. Moreover, increased IL-10 could inhibit and block CD8 T cell responses [58]. This might be another explanation for present findings that the majority of cytokines showed no statistical difference. Our study maybe preliminarily refused the contribution of CD8 T cell derived cytokines to discriminate different TB infection status, however, we can’t draw a definite conclusion owing to small sample.

Our study represents an extension of the IGRA theory. IGRA plays a pivotal role in the diagnosis of TB infection and enjoys widespread adoption in China. Nevertheless, it falls short in distinguishing between ATB and LTBI. The identification of a superior biomarker capable of discerning between ATB and LTBI during the TB infection diagnosis process is not only a subject of profound interest but also an imperative clinical requirement. According to findings from Luminex assay, discernible disparities in IFN-γ secretion levels between ATB and LTBI cohorts were absent. In contrast, both IP-10 and IL-1Ra in the realm of TB infection diagnosis and IP-10 in the specific diagnosis of ATB, and even in the differentiation between ATB and LTBI, may demonstrate enhanced diagnostic performance.

In our clinical practice, we frequently encounter systemic lupus erythematosus patients (SLE) presenting with symptoms such as fever, fatigue, rash, and multiple serous cavity effusions during their treatment. In such situations, a positive result from the IGRA test raises questions about whether the patient’s SLE is actively flaring or if ATB has developed. Accurate differentiation is crucial because it dictates the course of action. If the patient’s SLE is indeed active, we consider increasing the glucocorticoid dosage. However, if ATB is the underlying issue, immediate anti-TB treatment is warranted. Misdiagnosis and inappropriate treatment can lead to serious consequences. In the context of multi-cytokine analysis, one cytokine such as IL-1Ra, much like IGRA results, indicates the presence of TB infection. Another cytokine such as IP-10, can distinguish between ATB and LTBI (or vice versa), guiding us in selecting the appropriate treatment strategy.

Our study is an exploratory study with a small sample size, and Luminex assay with more operation difficulty and economic cost than traditional IGRAs, therefore, further studies should be performed to confirm and identify the optimal biomarker or combination of biomarkers with proper detection methods to enhanced diagnostic capacity in clinical practice with a larger number of participants. Besides, we conducted a single-center study and thus single-center effects cannot be excluded, which could be further validated by multi-center studies in the future. Furthermore, the involvement of these cytokines in the pathogenesis of TB infection is worth making a profound study.

Taken together, our findings indicate that, in addition to conventional IFN-γ, QFT-Plus-based cytokines including TNF-α, IL-6, IP-10, IL-1Ra and CXCL-1 could serve as potential biomarkers to determine TB disease states. Especially, IL-1Ra and IP-10, may have the most tremendous potential to discriminate different TB infection status. IL-1Ra may be the most promising marker to differentiate TB infection from HC and IP-10 could serve as the most ideal index to differentiate ATB from non-ATB and LTBI.

### Electronic supplementary material

Below is the link to the electronic supplementary material.

Supplementary Material 1

Supplementary Material 2

Supplementary Material 3

## Data Availability

The datasets used and/or analyzed during the current study available from the corresponding author on reasonable request.
