# Supplementary material for: Multiple cytokine analysis based on QuantiFERON-TB gold plus in different tuberculosis infection status: an exploratory study
Source: BMC Infect Dis. 2024 Jan 2;24:28. doi: 10.1186/s12879-023-08943-0 (PMC10762904; doi:10.1186/s12879-023-08943-0)
Supplement: Supplementary file 1 — Supplementary Material 1 [file 12879_2023_8943_MOESM1_ESM.docx]

**Supplementary Table 1.** AUCs of IL-10 concentration in QFT-Plus TB2 minus corresponding TB1 antigen tubes for distinguishing different TB infection status.

| Parameter | AUC (95%CI) | *p* |
| --- | --- | --- |
| Diagnosing TB infection | 0.742 (0.583-0.901) | 0.017 |
| Diagnosing ATB | 0.669 (0.506-0.831) | 0.039 |
| Differentiating ATB from LTBI | 0.687 (0.510-0.864) | 0.062 |

AUC, area under the curve; CI, confidence interval.
