# Supplementary material for: Multiple cytokine analysis based on QuantiFERON-TB gold plus in different tuberculosis infection status: an exploratory study
Source: BMC Infect Dis. 2024 Jan 2;24:28. doi: 10.1186/s12879-023-08943-0 (PMC10762904; doi:10.1186/s12879-023-08943-0)
Supplement: Supplementary file 3 — Supplementary Material 3 [file 12879_2023_8943_MOESM3_ESM.docx]

**Supplementary Figure 2.** The levels of 10 cytokines in QFT-Plus TB1 antigen tubes with the levels in the corresponding Nil tubes subtracted in the ATB, LTBI, previous TB and HC groups (previous TB with IGRA+ was included in LTBI). *P < 0.05, **P < 0.01, ***P < 0.001, ****P < 0.0001. Bars represent mean values, and error bars represent SD.


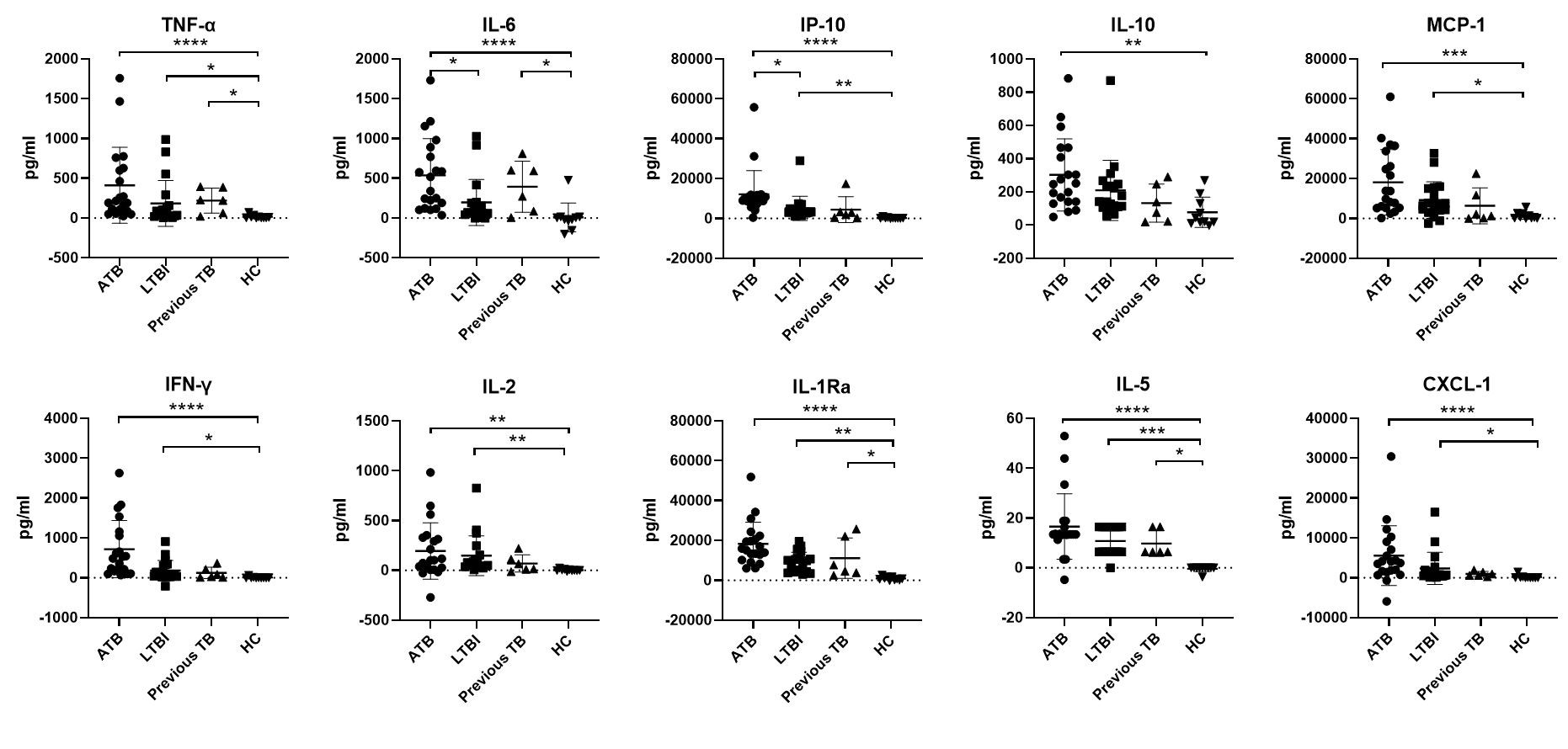


**Supplementary Figure 3.** The levels of 10 cytokines in QFT-Plus TB2 antigen tubes with the levels in the corresponding Nil tubes subtracted in the ATB, LTBI, previous TB and HC groups (previous TB with IGRA+ was included in LTBI). *P < 0.05, **P < 0.01, ***P < 0.001, ****P < 0.0001. Bars represent mean values, and error bars represent SD.


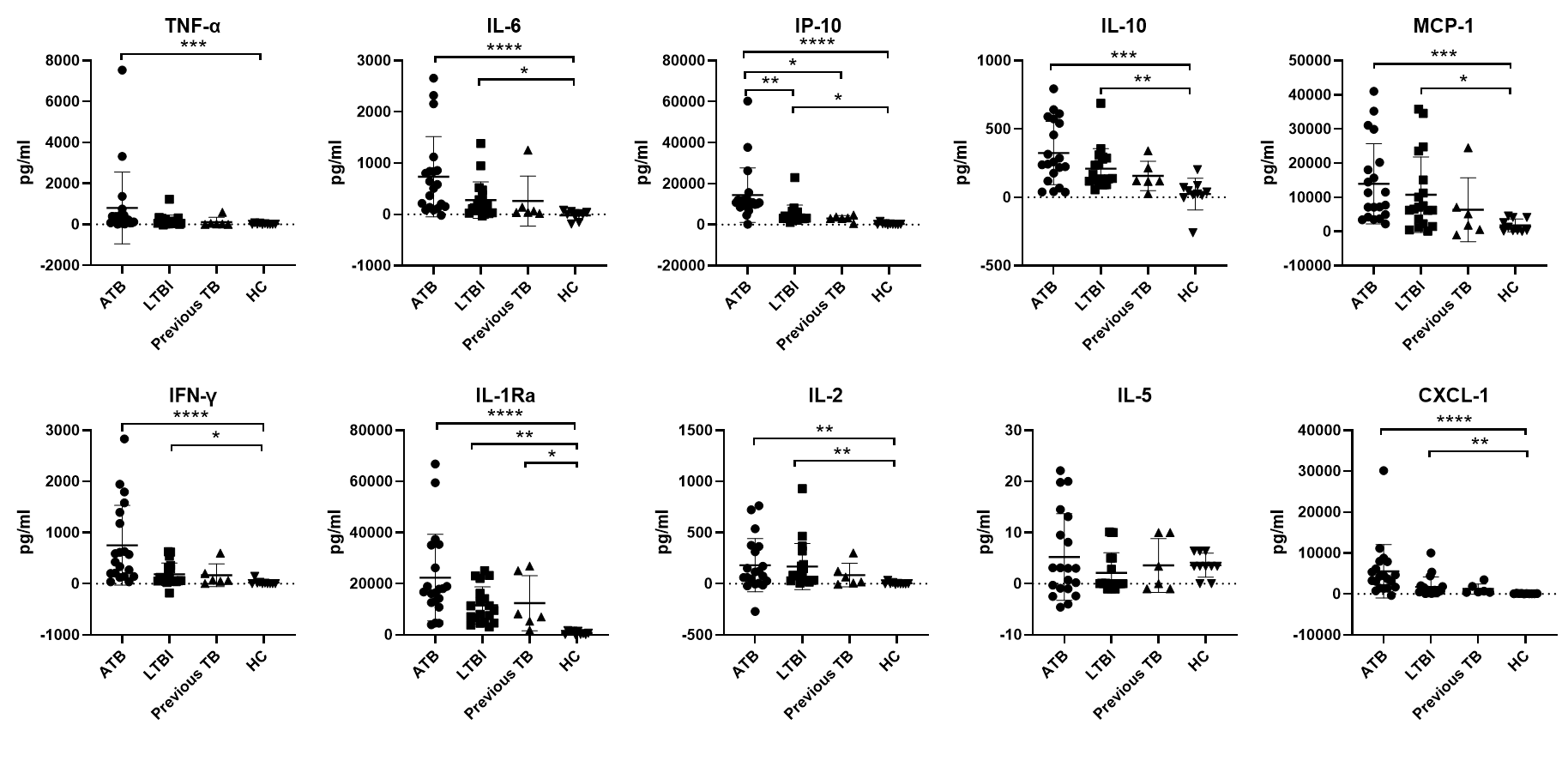


**Supplementary Figure 4.** The levels of 10 cytokines in QFT-Plus TB2 antigen tubes with the levels in the corresponding TB1 antigen tubes subtracted in the ATB, LTBI, previous TB and HC groups (previous TB with IGRA+ was included in LTBI). *P < 0.05. Bars represent mean values, and error bars represent SD.


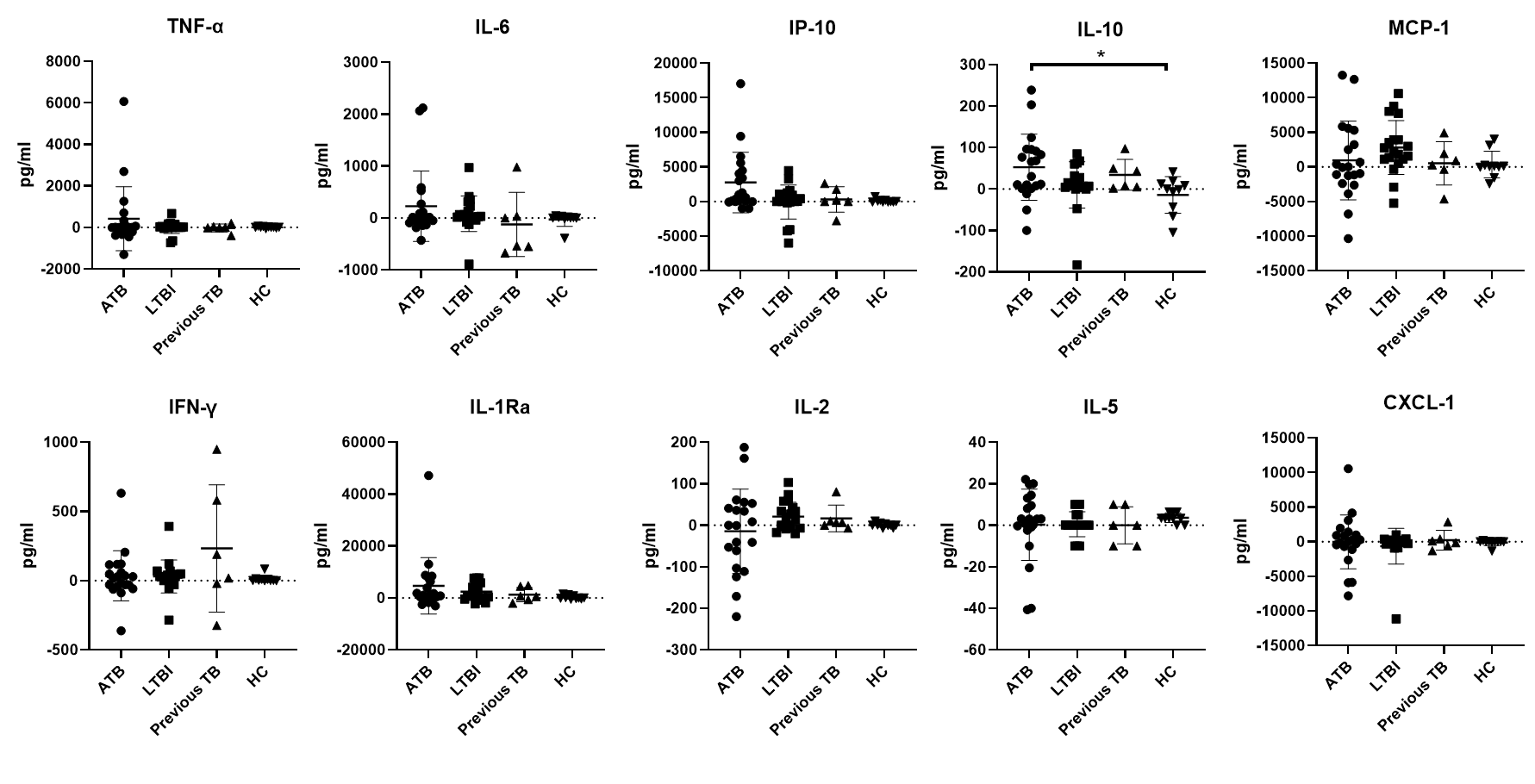


**Supplementary Figure 5.** ROC curve of cytokines in QFT-Plus TB1 and TB2 antigen tubes for distinguishing ATB from LTBI (previous TB with IGRA+ was included in LTBI). (A) ROC curve of the accuracy of cytokine levels stimulated by antigen in TB1 tube of QFT-Plus with the levels in the corresponding Nil tubes subtracted for differentiating ATB from LTBI. (B) ROC curve of the accuracy of cytokine levels stimulated by antigen in TB2 tube of QFT-Plus with the levels in the corresponding Nil tubes subtracted for differentiating ATB from LTBI. (C) ROC curve of the accuracy of cytokine levels stimulated by antigen in TB2 tube of QFT-Plus with the levels in the corresponding TB1 tubes subtracted for differentiating ATB from LTBI. Lines of different colors corresponding to matched cytokines.

**
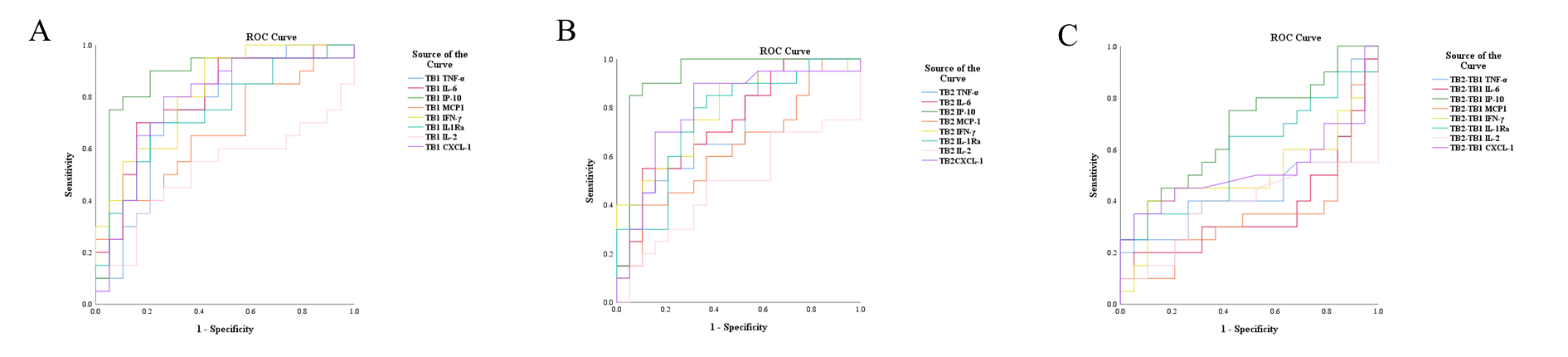
**

**Supplementary Table 2.** AUCs of cytokines in each antigen tube for distinguishing ATB from LTBI (previous TB with IGRA+ was included in LTBI).

| Parameter | TNF-α | IL-6 | IP-10 | MCP-1 | IFN-γ | IL-1Ra | IL-2 | CXCL-1 |
| --- | --- | --- | --- | --- | --- | --- | --- | --- |
| TB1-Nil | | | | | | | | |
| AUC (95%CI) | 0.745  (0.585-0.904) | 0.795  (0.651-0.938) | 0.871  (0.742-1.000) | 0.663  (0.492-0.834) | 0.816  (0.684-0.947) | 0.742  (0.585-0.899) | 0.513  (0.324-0.702) | 0.779  (0.625-0.933) |
| *p* | 0.009 | 0.002 | <0.001 | 0.081 | 0.001 | 0.010 | 0.888 | 0.003 |
| TB2-Nil | | | | | | | | |
| AUC (95%CI) | 0.734  (0.5782-0.891) | 0.742  (0.587-0.897) | 0.932  (0.840-1.000) | 0.629  (0.452-0.805) | 0.784  (0.640-0.928) | 0.761  (0.607-0.914) | 0.482  (0.295-0.668) | 0.796  (0.647-0.945) |
| *p* | 0.012 | 0.010 | <0.001 | 0.169 | 0.002 | 0.005 | 0.844 | 0.002 |
| TB2-TB1 | | | | | | | | |
| AUC (95%CI) | 0.459  (0.269-0.650) | 0.366  (0.182-0.549) | 0.684  (0.517-0.852) | 0.345  (0.164-0.525) | 0.500  (0.308-0.692) | 0.582  (0.399-0.764) | 0.391  (0.204-0.577) | 0.521  (0.327-0.715) |
| *p* | 0.663 | 0.152 | 0.049 | 0.097 | 1.000 | 0.384 | 0.244 | 0.822 |

AUC, area under the curve; CI, confidence interval.

**Supplementary Table 3.** The laboratory test results of smear acid-fast staining, culture, X-pert, TST, IGRA.

| Parameter. Positive/Total | ATB (n=20) | LTBI (n=15) | Previous TB (n=10) | HC (n=10) |
| --- | --- | --- | --- | --- |
| Smear acid-fast staining | 12/20 | / | / | / |
| Culture | 7/10 | / | / | / |
| X-pert | 20/20 | / | / | / |
| TST | 1/1 | 0/0 | / | 0/0 |
| IGRAs | 15/18 | 15/15 | 4/8 | 0/15 |
